# Effects of Pilates mat exercises on muscle strength and on pulmonary function in patients with cystic fibrosis*

**DOI:** 10.1590/S1806-37132014000500008

**Published:** 2014

**Authors:** Caroline Buarque Franco, Antonio Fernando Ribeiro, André Moreno Morcillo, Mariana Porto Zambon, Marina Buarque Almeida, Tatiana Rozov

**Affiliations:** State University at Campinas, Campinas, Brazil; Graduate Program in Child and Adolescent Health, State University at Campinas, Campinas, Brazil; Department of Pediatrics, State University at Campinas School of Medical Sciences, Campinas, Brazil; Graduate Program in Child and Adolescent Health, State University at Campinas, Campinas, Brazil; Department of Pediatric Pulmonology, Children’s Institute, University of São Paulo School of Medicine Hospital das Clínicas, São Paulo, Brazil; Graduate Program in Rehabilitation, Federal University of São Paulo, São Paulo, Brazil

**Keywords:** Cystic fibrosis, Muscle strength, Exercise movement techniques, Respiratory function tests

## Abstract

**OBJECTIVE:**

To analyze the effects of Pilates mat exercises in patients with cystic fibrosis (CF).

**METHODS:**

This was a clinical trial involving 19 CF patients recruited from either the CF Outpatient Clinic of the State University at Campinas *Hospital de Clínicas* or the Children's Institute of the University of São Paulo School of Medicine *Hospital das Clínicas*. All of the patients performed Pilates mat exercises for four months (one 60-min session per week). The variables studied (before and after the intervention) were respiratory muscle strength, MIP, MEP, FVC, and FEV_1_.

**RESULTS:**

After the intervention, MIP was significantly higher in the male patients (p = 0.017), as were MIP and MEP in the female patients (p = 0.005 and p = 0.007, respectively). There were no significant differences between the pre- and post-intervention values of FVC or FEV_1_, neither in the sample as a whole nor among the patients of either gender.

**CONCLUSIONS:**

Our results show that Pilates mat exercises have beneficial effects on respiratory muscle strength in CF patients.

## Introduction

The progression of lung disease in cystic fibrosis (CF) reduces the ability of individuals to participate in physical activity. Studies reveal that CF patients exhibit decreased muscle strength, which can contribute to fatigue during exercise and daily activities. In addition to decreased muscle mass and nutritional depletion, pulmonary function decline can increase exercise intolerance.^(^ 1 ^-^ 4 ^)^

Engaging in professionally-guided physical activity provides CF patients with numerous benefits, such as decreased dyspnea, bronchial clearance, improved body composition, maintenance of body development, decreased bone degradation with reduced muscle fatigue, greater IGF-I stimulation of anabolism, improved immune function, and decreased HR at rest.^(^ 1 ^-^ 8 ^)^

Progressive reduction in physical fitness, together with physical inactivity, starts a vicious cycle in which the worsening of dyspnea results in progressively decreasing physical exertion, resulting in a severely impaired quality of life. The use of Pilates mat exercises in the treatment of CF could interfere with this vicious cycle because these exercises involve controlling breathing and controlling contraction of the abdominal region.^(^ 8 ^-^ 13 ^)^

Since 2000, in the North-American fitness community, which has approximately five million practitioners, the Pilates method has progressively gained more followers. The technique is named after its creator, Joseph Pilates (1880-1967). Its first practitioners were almost exclusively dancers and athletes. Its approach is centered on taking an uninterrupted inhalation to prepare for the movement and subsequently using exhalation to execute it, with the abdomen being the power house; the instructor uses verbal commands, focusing on spine and lower limb alignment.^(^ 9 ^-^ 13 ^)^

Some studies have shown a relationship between physical fitness and respiratory muscle strength (RMS). Recent studies report that increased RMS improves the maintenance of breathing, demonstrating its importance for exercise tolerance in COPD. In order to measure the increase in RMS, a manometer is used because the technique involved is simple, practical, and noninvasive.^(^ 14 ^-^ 17 ^)^

Another significant assessment tool in CF is pulmonary function testing (PFT), the results of which are important for measurement of pulmonary impairment in studies of regular physical exercise programs. In PFT, measurement of FEV_1_ is a prognostic indicator of survival in CF patients. Patients with an FEV_1_ above 55% of predicted can practice physical exercises similarly to those practiced by healthy individuals.^(^ 2 ^,^ 3 ^,^ 18 ^)^

In view of the lack of studies evaluating the effects of Pilates mat exercises in CF patients, the primary objective of the present study was to analyze the pre- and post-intervention results for RMS by measuring MIP and MEP. A secondary objective was to use PFT to determine FVC and FEV_1_.

## Methods

This was a clinical trial involving 19 male and female patients aged 7-33 years and diagnosed with CF. Of those patients, 9 were treated at the CF Outpatient Clinic of the *Hospital de Clínicas da* *Universidade Estadual de Campinas* (HC-Unicamp, State University at Campinas *Hospital de Clínicas*), located in the city of Campinas, Brazil, and 10 were treated at the Children's Institute of the *Hospital das Clínicas da Faculdade de Medicina da Universidade de São Paulo *(HC-FMUSP, University of São Paulo School of Medicine *Hospital das Clínicas*), located in the city of São Paulo, Brazil.

Participants were followed regularly either at the *Associação Paulista de Assistência a Mucoviscidose* (São Paulo State Association for the Treatment of Mucoviscidosis), in the city of São Paulo, or at the outpatient clinic of the Unicamp Center for Pediatric Research, in the city of Campinas. Prior to the study onset, patients or their legal guardians gave written informed consent.

The present study did not affect the follow-up of patients at their facility of origin, which remained responsible for possible complications.

All of the patients performed Pilates mat exercises for 16 weeks (one 60-min session per week). There were two evaluations: the first one was performed before the Pilates intervention and the second one was performed on the sixteenth day after the intervention.

The inclusion criteria were as follows: having been diagnosed with CF; being 7 years of age or older; having an FEV_1_ > 30% of predicted; having abnormal results (sodium and chloride values greater than 60 mmol/L) in at least two sweat tests (samples of at least 100 mg), using the pilocarpine iontophoresis method, standardized by Gibson-Cooke; being clinically stable, i.e., having had no changes in symptoms and no changes in the maintenance treatment, in the last 30 days (data obtained from medical records); and being available to attend the sessions.

The exclusion criteria were as follows: being enrolled in a physical activity program; having missed ≥ 25% of the Pilates sessions; having *cor pulmonale*; having had episodes of exacerbation during the study period, the diagnostic criterion of an exacerbation being that used by Fuchs et al. (and consisting of 12 items; an episode of exacerbation is characterized by the presence of at least 4 items)^(^ 1 ^)^; having signs of decompensated lung disease (malaise, enhanced frequency and intensity of cough, or increased production of secretions) during the study period; having been hospitalized or having traveled during the study period; and having an SpO_2_ lower than normal before performing the Pilates mat exercises, which would make it impossible to perform the exercise program on the day of the treatment. Oximetry was performed at the beginning and at the end of all Pilates sessions.

### Pilates mat exercises

The Pilates mat program consisted of a weekly individual session lasting a maximum of 60 min, for 16 weeks. If the patient was under 18 years of age, the presence of a guardian was mandatory. All sessions were conducted by one of the researchers, who is Pilates trained.

The Pilates exercises used consisted of movements performed with the following equipment: a satin-finish vinyl foam mat; a rubber ball measuring 16.5 cm in diameter; Swiss balls measuring 55 and 65 cm in diameter; and an elastic tube.^(^ 9 ^,^ 10 ^)^

The activities were performed on a mat, with participants wearing no shoes, for better contact with the ground. The activity program included respiratory, postural, and abdominal exercises, as well as exercises for the trunk, upper limbs, and lower limbs. The movements were chosen based on the postural and musculoskeletal status of the patient, and the level of difficulty was gradually increased.^(^ 9 ^,^ 10 ^)^

On the first day of the treatment, the principles of the Pilates exercises were introduced. To facilitate learning, each exercise was first performed by the instructor and then by the patient. The principles consist of constant coordination between breathing and movement. Exhalation should be forced, and inhalation should be as natural as possible. The power house consists of isotonic (concentric and eccentric) contractions of the abnominal muscles, transverse abdominal muscle, multifidus muscle, and pelvic floor muscles, which are responsible for the body's static and dynamic stability. Breathing takes place simultaneously with the contraction of those structures.^(^ 9 ^-^ 13 ^)^

### Pulmonary function testing

All tests were performed at the Pulmonary Physiology Laboratory of the Unicamp Center for Pediatric Research, at the HC-Unicamp Pulmonary Function Testing Laboratory, or at the Pulmonary Function Laboratory of the HC-FMUSP Children's Institute. Patients were instructed on how to perform the test via demonstration of the appropriate technique, as guideline-recommended. At least three FVC curves were performed to ensure that maximal exertion and patient cooperation were achieved. The variables measured were FVC and FEV_1_. The analysis of the results considered the highest values of the measures of the acceptable curves, even if they were selected from different curves.^(^ 19 ^)^

### Measurement of MIP and MEP

A manometer (GER-AR, São Paulo, Brazil) calibrated in cmH_2_O was used to measure RMS. Patients were instructed to perform maximal inspiratory maneuvers, starting at RV, against the occluded valve, for measurement of MIP. For determination of MEP, patients performed maximal expiratory maneuvers, starting at TLC, against the occluded valve. Peak pressures were recorded. Three maneuvers were performed to measure each type of pressure, and the highest values (in cmH_2_O) were selected. Between measurements, patients were given a rest period of 1 min to allow for recovery from exertion. Measurements were performed with patients standing and using a nose clip; the nose clip prevented airflow through the nostrils during maximal exertion so that the airflow passed through the manometer circuit, thus avoiding incorrect results.^(^ 14 ^)^

### Statistical analysis

Statistical analysis was performed with the Statistical Package for the Social Sciences, version 16 (SPSS Inc., Chicago, IL, USA). The Wilcoxon test was used for paired comparison of two groups. In all cases, the level of significance was set at 5%.

### Ethical considerations

The present study was approved by the Research Ethics Committees of the Unicamp School of Medical Sciences (Protocol no. 967/2007; CAAE: 0707.0.146.000-07) and the FMUSP Department of Pediatrics (Protocol no. 007.40200832).

## Results

A total of 27 patients were invited to participate in the study. Of those, 8 were excluded because their clinical condition worsened during the data collection period or because they declined to participate in the study due to the great distance between their place of residence and the treatment center, given that some resided in cities or regions that were far from those centers. In addition, since most patients were enrolled in elementary, middle, or high school in the morning, many explained that they declined to participate in the study because they would have to miss classes during the study period.

Of the 19 participants, 12 were female. The mean age was 13.7 ± 7.4 years (range, 7-33 years). Demographic, anthropometric, spirometric data are described in Table 1. Table 2 shows the pre- and post-intervention values of MIP and MEP. After the intervention, MIP was significantly higher in the male patients (p = 0.017), as were MIP and MEP in the female patients (p = 0.005 and p = 0.007, respectively).

**Table 1:** Demographic, anthropometric, spirometric, and respiratory muscle strength data of 19 cystic fibrosis patients.a

Variable	Gender		Total
	Male	Female	
	(n = 7)	(n = 12)	(N = 19)
Age, years	13.6 ± 7.4	13.7 ± 7.4	13.7 ± 7.4
Weight, kg	36.50 ± 14.19	37.80 ± 12.95	37.30 ± 13.00
Height, m	1.43 ± 0.18	1.44 ± 0.15	1.40 ± 0.20
BMI, kg/m^2^	17.06 ± 2.98	17.57 ± 3.08	17.40 ± 3.00
FVC, % of predicted	78.12 ±17.55	80.00 ± 22.40	79.40 ± 20.40
FEV_1_, % of predicted	69.12 ± 18.51	69.49 ± 25.70	69.40 ± 23.00
MIP, cmH_2_O	77.85 ± 19.54	70.83 ±19.16	73.40 ± 19.10
MEP, cmH_2_O	67.85 ± 18.89	67.00 ± 14.53	67.40 ± 15.80

BMI: body mass index. aValues expressed as mean ± SD

**Table 2:** Distribution of the pre- and post-intervention (i.e., Pilates mat exercises) values of maximal inspiratory and expiratory pressures by gender.a

Gender	Variable	Pre-intervention	Post-intervention	p*
Male (n = 7)	MIP, cmH_2_O	77.85 ± 19.54	101.42 ± 22.67	0.017
	MEP, cmH_2_O	67.85 ± 18.89	85.00 ± 17.32	0.106
Female (n = 12)	MIP, cmH_2_O	70.83 ± 19.16	92.50 ± 17.25	0.005
	MEP, cmH_2_O	67.08 ± 14.53	81.66 ± 18.74	0.007

aValues expressed as mean ± SD. *Wilcoxon test

There were no significant differences between the pre- and post-intervention values of FVC or FEV_1_, neither in the sample as a whole nor among the patients of either gender (Table 3).

**Table 3:** Distribution of the pre- and post-intervention (i.e., Pilates mat exercises) values of spirometric parameters by gender.a

Gender	Variable	Pre-intervention	Post-intervention	p*
Male (n = 7)	FVC, % of predicted	78.16 ± 17.61	76.83 ± 13.30	0.463
	FEV_1_, % of predicted	69.16 ± 18.57	71.50 ± 18.39	0.598
Female (n = 12)	FVC, % of predicted	80.08 ± 22.42	81.41 ± 27.18	0.964
	FEV_1_, % of predicted	69.50 ± 25.74	71.50 ± 26.58	0.555

aValues expressed as mean ± SD. *Wilcoxon test

## Discussion

Chronic in course, CF is a disease that requires multidisciplinary treatment to improve quality of life and increase survival. The evidence supports the lasting benefits of physical exercise in this population. The effects of physical training programs are reached regardless of disease severity, i.e., even patients with more severe lung disease are able to improve their physical fitness.^(^ 18 ^,^ 20 ^,^ 21 ^)^

Given the progressive acceptance of the Pilates method and the lack of studies correlating this technique with CF, we evaluated the effects of Pilates mat exercises and the prospects for their future use in the routine treatment of CF patients.^(^ 3 ^,^ 5 ^-^ 7 ^)^

We found no published studies describing the effect of the Pilates method on RMS and, because we know the importance of RMS in reducing dyspnea and because muscle training in CF patients has not been fully explored, we examined exercise-related changes in RMS in these patients.

Results were separated by gender because of one study^(^ 4 ^)^ that reported differences in responses to training in men and women, with physical conditioning improving in both groups, but with performance being poorer in females, which can be attributed to differences in their physiological and morphological responses.

Pre- and post-intervention measurement of MIP and MEP revealed significant results in the sample as a whole. However, analysis of patients by gender showed that females had a significant increase in MEP, whereas males did not have a similar increase.

Because of its chronic nature with frequent lung infections, which produces the dyspnea-inactivity-dyspnea cycle, CF facilitates respiratory muscle fatigue. This condition impairs lung ventilation, limiting exercise tolerance. Studies involving other techniques and specific training exercises have confirmed benefits. Zanchet et al.,^(^ 16 ^)^ using a method known as thoracoabdominal rebalancing, reported increases in MIP and MEP, whereas Galvão,^(^ 17 ^)^ also evaluating RMS, found an increase in RMS, together with a reduction in the perception of dyspnea and an increase in exercise tolerance.

Research shows that the increase in respiratory muscle endurance results in improvements in the maintenance of breathing and in physical fitness. One group of authors^(^ 7 ^)^ reported increases in RMS after 4 weeks of training (25 min/day). The patients involved performed exercises for the upper limbs and respiratory muscles. According to those authors, the increase in this type of strength reduces muscle fatigue. Another study^(^ 18 ^)^ reported that individuals with lung disease generally use maximum breathing capacity during physical activity and have a greatly increased oxygen cost of breathing, which indicates airflow limitation.

Physical performance in CF correlates with lung disease severity, nutritional status, and peripheral muscle strength. Physical training programs to increase physical fitness and muscle strength in CF, as well as to facilitate expectoration and the execution of activities of daily living, have been investigated.^(^ 18 ^,^ 20 ^-^ 23 ^)^

In the present study, PFT results showed that there were no significant changes after the intervention. This finding confirms other results in the literature. In a systematic review,^(^ 21 ^)^ the authors reported the effectiveness of physical training programs in CF, and the 12 articles reviewed included anaerobic, aerobic, and resistance activities. The variables analyzed were PFT, aerobic capacity, strength, and quality of life. All of the studies found that physical training is beneficial in individuals with CF, and aerobic and resistance programs resulted in greater gains. No differences were found in PFT results, but those authors concluded that physical activity in CF patients resulted in a credit balance: maintenance of PFT values.^(^ 21 ^)^

Lung disease severity is one of the key factors in CF, and any intervention aimed at the long-term improvement or maintenance of pulmonary function would have significant implications for disease control and, subsequently, for survival.^(^ 8 ^)^

One group of authors^(^ 22 ^)^ developed a physical training program that provided benefits for pulmonary function. A 3-year randomized controlled trial of individualized home exercise in 65 children and adolescents was performed. The authors reported a reduction in the decline of FVC and FEV_1_. There is evidence of the importance of controlled long-term follow-up for achieving satisfactory results.

According to one group of authors,^(^ 23 ^)^ chronic pulmonary obstruction facilitates the decrease in FEV_1_, leading to hyperinflation and decreased breathing capacity during exercise. Children with CF develop a rapid breathing pattern during physical activity, and this makes their breathing more labored. The decreased muscle function (strength and endurance) observed in these patients leads to rapid respiratory muscle fatigue, which also contributes to reduced physical performance.

Recent studies have evaluated the effects of regular exercise on the life of adolescents with CF. However, there is still no consensus regarding the optimal training program. Most of these interventions are short in duration, with scarce longitudinal data, especially in the pediatric population. The lack of continuity of this type of training is quite common in this population, and maintaining it for long periods of time is a challenge, even if there is awareness of the positive impact that regular physical activity can provide. Family support is very important for patients to continue doing the exercises proposed by the clinical staff.

There is a need for more information about the effects of these types of intervention on the follow-up of CF patients. Controlled longitudinal studies are needed to determine the effects of regular physical activity on disease progression, analyzing the results on patient physical condition and comparing methods of longitudinal aerobic, anaerobic, or aerobic/anaerobic training. Detailed information regarding protocols, such as exercise intensity, exercise duration, and rest period, is important for professionals who work primarily with children with CF.

Methods involving careful assessment of physical fitness and clinical status show that most CF patients can engage in regular physical activity. It is up to the professionals involved in the treatment to provide information to patients and their parents, in order to ensure the adoption of appropriate exercise habits for maintaining and improving health.^(^ 4 ^-^ 8 ^,^ 18 ^-^ 23 ^)^

In conclusion, our results show that Pilates mat exercises have beneficial effects on RMS. These findings point to some considerations. There is a need to investigate large samples with a control group. It would be ideal for future research to increase the duration of the intervention and compare results after discontinuation of exercise training.
