# Supplementary material for: Effects of Pilates mat exercises on muscle strength and on pulmonary function in patients with cystic fibrosis
Source: J Bras Pneumol. 2014 Sep-Oct;40(5):521–7. doi: 10.1590/S1806-37132014000500008 (PMC4263333; doi:10.1590/S1806-37132014000500008)
Supplement: Translation

# Translation: Efeitos do método Pilates na força muscular e na função pulmonar de pacientes com fibrose cística*

**Authors:** Caroline Buarque Franco, Antonio Fernando Ribeiro, André Moreno Morcillo, Mariana Porto Zambon, Marina Buarque Almeida, Tatiana Rozov

**Affiliations:** Universidade Estadual de Campinas, Campinas (SP) Brasil; Programa de Pós-Graduação em Saúde da Criança e do Adolescente, Universidade Estadual de Campinas, Campinas (SP) Brasil; Departamento de Pediatria, Faculdade de Ciências Médicas, Universidade Estadual de Campinas, Campinas (SP) Brasil; Programa de Pós-Graduação em Saúde da Criança e do Adolescente, Universidade Estadual de Campinas, Campinas (SP) Brasil; Unidade de Pneumologia Pediátrica, Instituto da Criança, Faculdade de Medicina, Universidade de São Paulo, São Paulo (SP) Brasil; Programa de Pós-Graduação em Reabilitação, Universidade Federal de São Paulo, São Paulo (SP) Brasil

**Keywords:** Fibrose cística, Força muscular, Técnicas de exercício e de movimento, Testes de função respiratória

## Abstract

**OBJETIVO:**

Analisar os efeitos do método Pilates em pacientes com fibrose cística (FC).

**MÉTODOS:**

Foi realizado um ensaio clínico, casuística de 19 pacientes com FC. Os pacientes foram recrutados no Ambulatório de FC do Hospital de Clínicas da Universidade Estadual de Campinas e no Instituto da Criança do Hospital das Clínicas da Faculdade de Medicina da Universidade de São Paulo. Todos os pacientes foram submetidos a uma sessão semanal de Pilates de 60 min durante quatro meses. As variáveis estudadas, antes e após a intervenção, foram força muscular respiratória, PImáx, PEmáx, CVF e VEF_1_.

**RESULTADOS:**

Houve um aumento significativo na PImáx nos pacientes do sexo masculino (p = 0,017), enquanto houve aumentos significativos na PImáx e PEmáx nos pacientes do sexo feminino (p = 0,005 e p = 0,007, respectivamente) após a intervenção. Não houve diferenças significativas nos valores de CVF e VEF_1_ antes e após a intervenção no grupo total de participantes, nem nos subgrupos em relação ao gênero.

**CONCLUSÕES:**

Os resultados deste estudo mostraram os efeitos benéficos da aplicação do método Pilates na força muscular respiratória nos pacientes estudados. (Registro Brasileiro de Ensaios Clínicos - ReBEC; número de identificação RBR-86vp8x [http://www.ensaiosclinicos.gov.br])

## Introdução

A progressão da doença pulmonar na fibrose cística (FC) reduz a habilidade para a participação em atividades físicas. Estudos revelam que pacientes com FC apresentam diminuição da força muscular, o que pode contribuir para a fadiga durante o exercício e as atividades diárias. Além da diminuição de massa muscular e da depleção nutricional, o declínio da função pulmonar também pode acentuar a intolerância ao exercício.^(^ 1 ^-^ 4 ^)^

A inclusão de atividades físicas, profissionalmente orientadas, proporciona numerosos benefícios, como redução da dispneia, desobstrução brônquica, melhora da composição corporal, manutenção do desenvolvimento ósseo, diminuição da degradação proteica com redução da fadiga muscular, maior estímulo do IGF-I para o anabolismo, melhoria da função imunológica e diminuição da FC de repouso.^(^ 1 ^-^ 8 ^)^

A diminuição progressiva do condicionamento físico, aliado à inatividade, inicia um ciclo vicioso no qual a piora da dispneia resulta em esforços físicos cada vez menores, com grave comprometimento da qualidade de vida. A proposta da realização do método Pilates na FC pode interferir nesse ciclo por realizar o controle da respiração e da contração da região abdominal.^(^ 8 ^-^ 13 ^)^

A partir de 2000, na comunidade de *fitness *norte-americana, com aproximadamente cinco milhões de praticantes, o método Pilates vem ganhando progressivamente novos adeptos. A técnica recebe esse nome por fazer referência a seu criador, Joseph Pilates (1880-1967). Os primeiros praticantes da técnica foram quase exclusivamente dançarinos e atletas. Sua abordagem concentra-se na realização contínua da inspiração ao iniciar o movimento, seguida pela expiração ao executá-lo, tendo o abdômen como centro de força; o instrutor utiliza comandos verbais focando o alinhamento da coluna e dos membros inferiores.^(^ 9 ^-^ 13 ^)^

Alguns estudos observaram a relação do condicionamento físico com a força muscular respiratória (FMR). Trabalhos recentes evidenciam que o aumento da FMR melhora a sustentação da respiração, demonstrando sua importância na tolerância aos esforços na DPOC. Para acompanhar o ganho da FMR, utiliza-se um manovacuômetro na avaliação por essa ser uma técnica simples, prática e não invasiva.^(^ 14 ^-^ 17 ^)^

Outra avaliação considerável na FC é a prova de função pulmonar (PFP), cujos resultados são importantes em estudos sobre programas de exercícios físicos regulares com o intuito de acompanhar o comprometimento pulmonar. Na PFP, a mensuração do VEF_1_ é um indicador do prognóstico de sobrevida desses pacientes. Pacientes com VEF_1_ acima dos 55% do previsto estão aptos para a prática de exercícios físicos similares àqueles praticados por indivíduos saudáveis.^(^ 2 ^,^ 3 ^,^ 18 ^)^

Diante da ausência de publicações que avaliem os efeitos do método Pilates em pacientes com FC, o presente estudo teve como objetivo primário analisar os resultados, antes e após a intervenção, do comportamento da FMR, através da medição da PImáx e da PEmáx; como objetivo secundário, utilizamos a PFP para avaliar CVF e VEF_1_.

## Métodos

Realizou-se um ensaio clínico em 19 pacientes com diagnóstico de FC, com idade variando de 7 a 33 anos, de ambos os sexos; 9 desses pacientes eram atendidos no Ambulatório de Assistência ao Paciente com FC do Hospital de Clínicas (HC) da Universidade Estadual de Campinas (Unicamp), localizado na cidade de Campinas (SP), e 10 eram atendidos no Instituto da Criança do HC da Faculdade de Medicina da Universidade de São Paulo (FMUSP), localizado na cidade de São Paulo (SP).

Os participantes foram acompanhados regularmente na Associação Paulista de Assistência a Mucoviscidose, na cidade de São Paulo, ou no ambulatório do Centro de Investigação em Pediatria da Unicamp, na cidade de Campinas. Antes da realização do estudo, os pacientes ou seus responsáveis assinaram um termo de consentimento livre e esclarecido.

A pesquisa não interferiu no acompanhamento dos participantes nos seus serviços de origem, os quais permaneceram responsáveis por possíveis intercorrências.

Todos os pacientes foram submetidos a sessões do método Pilates uma hora por dia, uma vez por semana, durante 16 semanas. Foram realizadas duas avaliações: a primeira antes da intervenção e a segunda no 16º dia após a intervenção com Pilates.

Os critérios de inclusão do presente estudo foram os seguintes: ter diagnóstico de FC; ter idade ≥ 7 anos; apresentar VEF_1_ > 30% do previsto; apresentar resultados alterados (sódio e cloro com valores maiores que 60 mmol/L) em pelo menos dois testes de suor (amostras com ao menos 100 mg), obtidos pelo método da iontoforese por pilocarpina, padronizado por Gibson-Cooke; apresentar estabilidade clínica da doença, definida por ausência de mudanças nos sintomas e ausência de alterações no tratamento de manutenção, nos últimos 30 dias (esses dois últimos critérios foram obtidos pelo acompanhamento dos prontuários); e ter disponibilidade em comparecer às sessões.

Os critérios de exclusão foram os seguintes: estar inserido em algum programa de atividade física; ter tido ≥ 25% de faltas nas sessões de Pilates; apresentar *cor pulmonale;* apresentar episódios de exacerbações durante o período de estudo, de acordo com o critério de Fuchs et al.,(composto por 12 itens; a presença de pelo menos 4 itens caracteriza o episódio de exacerbação)^(^ 1 ^)^; apresentar sinais de descompensação da doença pulmonar durante período de estudo (mal-estar, aumento da frequência e intensidade da tosse ou aumento da produção de secreções); ter sido internado ou ter viajado durante o período de estudo; e apresentar SpO_2_ abaixo do normal antes da realização do Pilates, o que impossibilitaria a realização do programa de exercícios no dia do atendimento. A oximetria foi realizada no início e no término de todas as sessões de Pilates.

### Método Pilates

O programa de exercícios pelo método Pilates consistiu em uma sessão semanal individual, no tempo máximo de 60 min, por 16 semanas. No caso de o paciente ter menos de 18 anos de idade, era obrigatória a presença de um responsável. Todas as sessões foram realizadas por uma das pesquisadoras, que apresenta formação para a utilização do método.

Os exercícios aplicados fazem parte do método Pilates de solo com bola suíça, que consiste na aplicação de movimentos utilizando os seguintes materiais: colchonete de espuma vinílica acetinada, bola de borracha com 16,5 cm de diâmetro, bolas suíças com 55 e 65 cm de diâmetro e tubo elástico.^(^ 9 ^,^ 10 ^)^

As atividades foram realizadas sobre um colchonete, sem uso de calçados para um melhor contato com o solo. O programa de atividades apresentava exercícios respiratórios, posturais e abdominais, além de exercícios para o tronco, membros superiores e membros inferiores. Os movimentos foram escolhidos de acordo com a condição postural e musculoesquelética do paciente, com aumentos graduais do nível de dificuldade.^(^ 9 ^,^ 10 ^)^

No primeiro dia de atendimento, os princípios do método Pilates eram repassados. O exercício era demonstrado pela instrutora para facilitar o aprendizado e, em seguida, o paciente executava-o. Os princípios consistem na realização da respiração sempre coordenada com o movimento. A expiração deve ser forçada, e a inspiração deve ser o mais natural possível. O *power house* (centro de força) consiste em contrações isotônicas (concêntricas e excêntricas) realizadas pelos músculos abdominais, transverso abdominal, multifidos e músculos do assoalho pélvico, que são responsáveis pela estabilização estática e dinâmica do corpo. A respiração era realizada juntamente com a contração dessas estruturas.^(^ 9 ^-^ 13 ^)^

### Provas de função pulmonar

Os exames foram realizados no Laboratório de Fisiologia Pulmonar LAFIP no Centro de Investigação em Pediatria da Unicamp, no Laboratório de Prova de Função Pulmonar do HC-Unicamp ou no Laboratório de Função Pulmonar do Instituto da Criança do HC-FMUSP. Os pacientes foram instruídos para a execução do teste através da demonstração da técnica adequada, seguindo a padronização. Ao menos três curvas aceitáveis da CVF foram realizadas com o objetivo de assegurar que o esforço máximo e a cooperação do paciente fossem obtidas. As variáveis aferidas foram a CVF e o VEF_1._ Para a análise dos resultados, foram considerados os maiores valores das medidas das curvas consideradas aceitáveis, mesmo que tenham sido selecionados de curvas diferentes.^(^ 19 ^)^

### Avaliação da PImáx e PEmáx

Para avaliar a FMR foi utilizado um manovacuômetro (GER-AR, São Paulo, Brasil) calibrado em cmH_2_O. O paciente foi orientado a realizar uma inspiração máxima a partir do VR contra a válvula ocluída para a mensuração da PImáx. Para a determinação da PEmáx, o paciente realizou uma expiração máxima a partir da CPT contra a válvula ocluída. Foram registradas as pressões de pico. Para ambas as pressões foram realizadas três manobras, sendo os maiores valores, registrados em cmH_2_O, selecionados. Entre cada medida, respeitou-se um intervalo de 1 min a fim de que o paciente se recuperasse do esforço realizado. As medições foram realizadas com o paciente em pé e utilizando um clipe nasal; a passagem de ar pelas narinas era evitada durante o esforço máximo para que o ar fosse conduzido pelo circuito do manovacuômetro, evitando-se assim a obtenção de valores incorretos.^(^ 14 ^)^

### Análise estatística

A análise estatística foi feita com o auxílio do programa *Statistical Package for the Social Sciences*, versão 16 (SPSS Inc., Chicago, IL, EUA). Para a comparação da distribuição de dois grupos pareados empregou-se o teste de Wilcoxon. Em todos os casos, adotou-se o nível de significância de 5%.

### Considerações éticas

O estudo foi aprovado pelos comitês de ética em pesquisa da Faculdade de Ciências Médicas da Unicamp (protocolo no. 967/2007; CAAE: 0707.0.146.000-07) e do Departamento de Pediatria da FMUSP (protocolo no. 007.40200832).

## Resultados

Foram convidados para participar do estudo 27 pacientes. Desses, 8 foram excluídos por piora do quadro clínico durante o período da coleta de dados ou pela recusa em participar do estudo devido à grande distância entre o local de residência e o centro de tratamento, pois alguns residiam em outras cidades ou em outras regiões distantes desses centros. Além disso, como a maioria dos pacientes encontrava-se matriculada no ensino fundamental ou médio no período matutino, muitos justificaram a recusa em participar do estudo devido ao fato de terem que faltar às aulas durante o período do estudo.

Dos 19 participantes, 12 eram do gênero feminino. A média de idade foi de 13,7 ± 7,4 anos (variação, 7-33 anos). Dados demográficos, antropométricos e espirométricos estão descritos na Tabela 1. Na Tabela 2, estão apresentados os resultados de PImáx e PEmáx dos participantes antes e após a intervenção. Houve um aumento significativo na PImáx nos pacientes do sexo masculino (p = 0,017) após a intervenção, enquanto houve aumentos significativos na PImáx e PEmáx nos pacientes do sexo feminino (p = 0,005 e p = 0,007, respectivamente).

**Tabela 1:** Dados demográficos, antropométricos, espirométricos e de força muscular respiratória de 19 pacientes com fibrose cística.a

Variáveis	Gênero		Total
	Masculino	Feminino	
	(n = 7)	(n = 12)	(N = 19)
Idade, anos	13,6 ± 7,4	13,7 ± 7,4	13,7 ± 7,4
Peso, kg	36,50 ± 14,19	37,80 ± 12,95	37,30 ± 13,00
Altura, m	1,43 ± 0,18	1,44 ± 0,15	1,40 ± 0,20
IMC, kg/m^2^	17,06 ± 2,98	17,57 ± 3,08	17,40 ± 3,00
CVF, % do previsto	78,12 ±17,55	80,00 ± 22,40	79,40 ± 20,40
VEF_1_, % do previsto	69,12 ± 18,51	69,49 ± 25,70	69,40 ± 23,00
PImáx, cmH_2_O	77,85 ± 19,54	70,83 ±19,16	73,40 ± 19,10
PEmáx, cmH_2_O	67,85 ± 18,89	67,00 ± 14,53	67,40 ± 15,80

IMC: índice de massa corpórea

a Valores expressos em média ± dp.

**Tabela 2:** Distribuição dos valores das pressões inspiratórias e expiratórias máximas antes e após a intervenção com Pilates por gênero.a

Gênero	Variáveis	Intervenção		p*
		Antes	Após	
Masculino (n = 7)	PImáx, cmH_2_O	77,85 ± 19,54	101,42 ± 22,67	0,017
	PEmáx, cmH_2_O	67,85 ± 18,89	85,00 ± 17,32	0,106
Feminino (n = 12)	PImáx, cmH_2_O	70,83 ± 19,16	92,50 ± 17,25	0,005
	PEmáx, cmH_2_O	67,08 ± 14,53	81,66 ± 18,74	0,007

a Valores expressos em média ± dp

* Teste de Wilcoxon.

Em relação às variáveis espirométricas, não houve diferenças significativas nos valores de CVF e VEF_1_ antes e após a intervenção no grupo total de participantes, nem nos subgrupos em relação ao gênero (Tabela 3). 

**Tabela 3:** Distribuição dos valores espirométricos estudados antes e após a intervenção com Pilates por gênero.a

Gênero	Variáveis	Intervenção		p*
		Antes	Após	
Masculino (n = 7)	CVF, % do previsto	78,16 ± 17,61	76,83 ± 13,30	0,463
	VEF_1_, % do previsto	69,16 ± 18,57	71,50 ± 18,39	0,598
Feminino (n = 12)	CVF, % do previsto	80,08 ± 22,42	81,41 ± 27,18	0,964
	VEF_1_, % do previsto	69,50 ± 25,74	71,50 ± 26,58	0,555

a Valores expressos em média ± dp

* Teste de Wilcoxon.

## Discussão

A FC é uma doença crônica que demanda terapias multidisciplinares, visando a melhoria da qualidade de vida e maior sobrevida. As evidências comprovam benefícios duradouros com a realização de exercícios físicos nessa população. Os efeitos de programas de treinamento físico são alcançados independentemente da gravidade da doença, ou seja, mesmo pacientes que apresentam uma condição pulmonar mais grave são capazes de melhorar seu condicionamento físico.^(^ 18 ^,^ 20 ^,^ 21 ^)^

Diante da aceitação progressiva de praticantes do método Pilates e da ausência de estudos que relacionem essa técnica com a FC, avaliamos os seus efeitos e sua futura aplicação na rotina de tratamento desses pacientes.^(^ 3 ^,^ 5 ^-^ 7 ^)^

Não encontramos publicações na literatura que mostrem a ação do método Pilates sobre a FMR e, por sabermos da importância da FMR na diminuição da dispneia, e como o treinamento muscular em pacientes com FC não foi totalmente explorado, acompanhamos a evolução da FMR nesses pacientes.

A separação dos resultados entre os gêneros foi realizada baseando-se em um estudo^(4) ^que mostrou diferenças nas respostas ao treinamento em homens e mulheres, com melhora do condicionamento físico em ambos os grupos, mas com um menor desempenho no gênero feminino, que pode ser atribuído às diferenças em suas respostas fisiológicas e morfológicas.

A avaliação da PImáx e PEmáx antes e após a intervenção mostrou resultados significativos no grupo total de pacientes; porém, ao avaliarmos os pacientes de acordo com gênero, o sexo feminino apresentou um aumento significativo da PEmáx, enquanto o sexo masculino não obteve ganhos semelhantes.

A FC, por seu caráter crônico com infecções pulmonares frequentes, o que gera o ciclo dispneia-sedentarismo-dispneia, facilita a fadiga da musculatura respiratória. Essa condição agrava a ventilação pulmonar, limitando a tolerância aos exercícios. Estudos com outras técnicas e exercícios específicos de treinamentos comprovaram benefícios. Zanchet et al.,^(^ 16 ^)^ aplicando o método de reequilíbrio toracoabdominal, demonstraram ganhos na PImáx e PEmáx, enquanto Galvão,^(^ 17 ^)^ também avaliando a FMR, evidenciou ganho de força dessa musculatura, com redução da sensação de dispneia e aumento da tolerância aos exercícios.

Pesquisas apontam que o aumento da capacidade desses músculos proporciona melhoras na sustentação da respiração e no condicionamento físico. Um grupo de autores^(7) ^demonstrou ganhos na FMR após 4 semanas de treinamento (25 min/dia). Os pacientes envolvidos realizaram exercícios para os membros superiores e músculos da respiração. Segundo os autores, o aumento desse tipo de força reduz a fadiga muscular. Em outro estudo,^(18) ^demonstrou-se que indivíduos com doença pulmonar geralmente utilizam a sua máxima capacidade respiratória durante as atividades físicas, sendo o custo de oxigênio muito maior para a respiração, o que indica limitação ventilatória.

O desempenho físico na FC apresenta uma relação com a gravidade da doença pulmonar, o estado nutricional e a força muscular periférica. Treinamentos físicos são investigados para aumentar o condicionamento físico e o ganho da resistência muscular, assim como para a facilitação da expectoração e da execução das atividades de vida diária na FC.^(^ 18 ^,^ 20 ^-^ 23 ^)^

Os resultados da PFP evidenciaram a ausência de mudanças significativas após a intervenção. Esse dado confirma os achados da literatura. Em uma revisão sistemática,^(^ 21 ^)^ os autores mostraram a eficácia dos programas de treinamento físico na FC, e os 12 artigos revisados incluíam atividades anaeróbicas, aeróbicas e de resistência. As variáveis analisadas foram PFP, capacidade aeróbica, força e qualidade de vida. Todos os estudos verificaram que o treinamento físico é benéfico em indivíduos com FC, e os programas aeróbicos e de resistência resultaram em ganhos maiores. Não foram encontradas diferenças nos exames de PFP, mas os autores concluíram que houve um saldo positivo pela influência da realização da atividade física na FC, que foi a preservação e a manutenção dos valores da PFP.^(^ 21 ^)^

A gravidade da doença pulmonar é um dos fatores determinantes na FC, e qualquer intervenção visando melhorias ou a preservação da função pulmonar a longo prazo teria implicações fundamentais no controle da doença e, subsequentemente, na sobrevivência.^(^ 8 ^)^

Um grupo de autores^(22) ^desenvolveu um programa de treinamento físico que trouxe benefícios para a função pulmonar. Durante 3 anos, foi desenvolvido um estudo controlado, randomizado e individualizado envolvendo 65 crianças e adolescentes que realizavam um protocolo em seus domicílios. Os autores relataram uma redução do declínio de CVF e VEF_1_. Há evidências da importância da realização de acompanhamentos controlados em longo prazo para o alcance de resultados satisfatórios.

Segundo um grupo de autores,^(^ 23 ^)^ a obstrução pulmonar crônica facilita a diminuição do VEF_1_, gerando hiperinsuflação e diminuição da capacidade respiratória durante os exercícios. Crianças com FC desenvolvem um padrão de respiração rápido durante as atividades físicas, tornando a respiração mais trabalhosa. A função muscular reduzida (força e resistência) observada nesses pacientes leva a rápida fadiga muscular respiratória, o que também contribui na redução do desempenho físico.

Trabalhos recentes têm avaliado a interferência do exercício regular na vida de adolescentes com FC; entretanto, ainda não existe consenso sobre o programa de treinamento ideal. Grande parte desse tipo intervenção é de curta duração, com dados longitudinais escassos, especialmente na população pediátrica. A falta de continuidade desses regimes é bem comum nessa população e sustentá-los por longos períodos de tempo é um desafio, mesmo que haja o conhecimento do impacto positivo que a prática da atividade física regular pode proporcionar. O apoio dos familiares é muito importante para a continuidade dos exercícios propostos pela equipe de atendimento.

Há a necessidade de mais informações sobre os efeitos desses tipos de intervenção no acompanhamento de pacientes com FC. Estudos longitudinais e controlados são necessários para verificar os efeitos das atividades físicas regulares na progressão da doença, analisando os resultados na condição física e comparando métodos de treinamento longitudinais aeróbicos, anaeróbicos ou a combinação de ambos. Informações detalhadas de protocolos, como a intensidade dos exercícios, sua duração e tempo de repouso, são importantes para os profissionais que trabalham principalmente com crianças com FC.

Métodos com avaliações criteriosas da aptidão física e do estado clínico mostram que grande parte dos pacientes com FC se encontra apta para a prática de atividade física regular. Cabe aos profissionais envolvidos no atendimento fornecer informações aos pacientes e aos pais, com o intuito de garantir a adoção de hábitos adequados de exercício físico para a manutenção e melhora da saúde.^(^ 4 ^-^ 8 ^,^ 18 ^-^ 23 ^)^

Em conclusão, os resultados do presente estudo mostraram os efeitos benéficos da aplicação do método Pilates na FMR. Frente a esses achados, algumas considerações devem ser feitas. Casuísticas maiores e a inclusão de um grupo controle deveriam ser investigadas. Prolongar o tempo de intervenção e comparar os resultados após a suspensão desse tipo de treinamento seria ideal para futuras pesquisas.
